# Reframing prevention: A clinician’s perspective

**DOI:** 10.1080/13814788.2025.2549809

**Published:** 2025-08-22

**Authors:** Paulo Santos

**Affiliations:** Faculty of Medicine-MEDCIDS, RISE-Health@RISE Alameda Hernani Monteiro, Porto University, Porto, Portugal

Dear Editor

The EUROPREV position paper offers a thoughtful foundation for improving preventive strategies in general practice and family medicine [1]. Its focus on caution and restraint is rooted in legitimate concerns about overdiagnosis and harm. Yet, such a posture invites reflection: can too much hesitancy in prevention become a form of clinical inertia?

First, delays in preventive action may result not in safety, but in lost opportunity. In cancer screening, like lung or prostate cancer, early detection can transform outcomes [2]. While epidemiology parses population-level risk, the individual in front of us is not a statistical abstraction. In practice, general consultations are not arenas for statistical purity but sites of clinical discernment, empathy, and timely care. The epidemiological lens is essential, but it must not obscure the needs of the person sitting across from us.

Evidence-based medicine, too, must be seen in its full dimension. As Sackett reminds us, it is not merely about trial data, but the integration of clinical expertise, patient preferences, and scientific evidence [3]. Much of general practice functions with this broader understanding, where intuition, experience, and context inform meaningful decisions, even when data is incomplete. Prevention is not a technical checklist; it is a human act.

Second, the paper rightly positions primary care at the heart of prevention, but the landscape is wider. Effective prevention demands collaboration across all levels of care, including community, specialists, and hospitals, and alignment with public health systems. Crucially, the social determinants of health, including housing, education, and income, are not environmental backdrops but central drivers of illness and wellness [4]. A siloed approach limits our reach. Prevention must be systemic, not solitary.

Health literacy is essential to this vision. Prevention is not simply the absence of illness: it is the presence of agency, understanding, and informed choice. Equipping individuals to assess and act on health information is vital, even when the desired outcome is to remain well [5]. Preventive care must empower, not just advise.

Third, the principle of starting low and going slow holds value when viewed as a call for sustainability. However, caution should not become paralysis. Inertia can kill just as excess can harm. The challenge is to act wisely, compassionately, and without fear. Overdiagnosis is real, but so is the missed diagnosis. Doing nothing may protect us from statistical regret, but it will not protect patients from the consequences of delay [6].

Finally, the ethical stance of the position paper seems to lean heavily towards non-maleficence. While important, it must be balanced with beneficence. We are not here merely to avoid harm: we are here to make good things happen, carefully and responsibly. Excessive fear can silence medicine, and silence can leave suffering untreated, opportunities wasted, and lives lost.

The EUROPREV paper succeeds in opening the debate. Let us take it further: towards a model of prevention that is assertive yet thoughtful, inclusive yet personalised, and guided not only by what might go wrong but by all that could go right.
